# Asymptomatic esophageal perforation discovered after elective cardiac surgery: the importance of clinical awareness

**DOI:** 10.1186/s13019-024-02551-y

**Published:** 2024-02-03

**Authors:** N. A. Hasami, W. W. L. Li, T. Smith, A. F. T. M. Verhagen, K. Ko, R. H. Heijmen

**Affiliations:** Department of Cardiothoracic Surgery, RadboudUMC, Geert Grooteplein Zuid 10, 6525 GA Nijmegen, The Netherlands

## Abstract

Transesophageal echocardiography (TEE) has become an indispensable part of cardiothoracic surgery at present and is considered to be a safe procedure, rarely associated with complications. However, TEE may cause serious and life threatening complications, as presented in this case report. We describe a patient who developed an empyema after elective cardiac surgery due to an esophageal perforation caused by TEE, without any clinical symptoms. Risk factors for TEE-related complications, identified in recent literature, will be discussed as well as the remarkable absence of clinical symptoms in this particular patient.

## Introduction

Transesophageal echocardiography (TEE) has become an indispensable part of perioperative care in cardiac surgery. The use of TEE is potentially valuable in all phases of the perioperative process: pre-operatively for detailed analysis of cardiac and/or valvular pathology, intra-operatively for guidance in anesthesia and surgery, and post-operatively on the Intensive Care for initial follow-up assessment. In general, TEE is considered to be safe, with a reported complication rate of 0.1–0.2% [1, 2]. However, it is important for clinicians to be aware that TEE can cause serious and life-threatening complications, for example upper gastrointestinal (GI) perforation and upper-GI bleeding. Other commonly reported complications are dental injuries, lip compression, oropharyngeal injuries and dysphagia/odynophagia [3].

We present a case of a patient who developed an empyema within a few days of elective cardiac surgery due to an asymptomatic distal esophageal perforation caused by TEE. The remarkable absence of complaints and the diagnostic challenges associated with this case will be discussed. Furthermore, the risk factors of TEE-related complications will be addressed. Ultimately the objective of this clinical presentation is to increase awareness for such rare complications and emphasize that, despite all benefits, a careful consideration of the indication for the use of TEE is required at all times.

## Case presentation

**Fig. 1:**
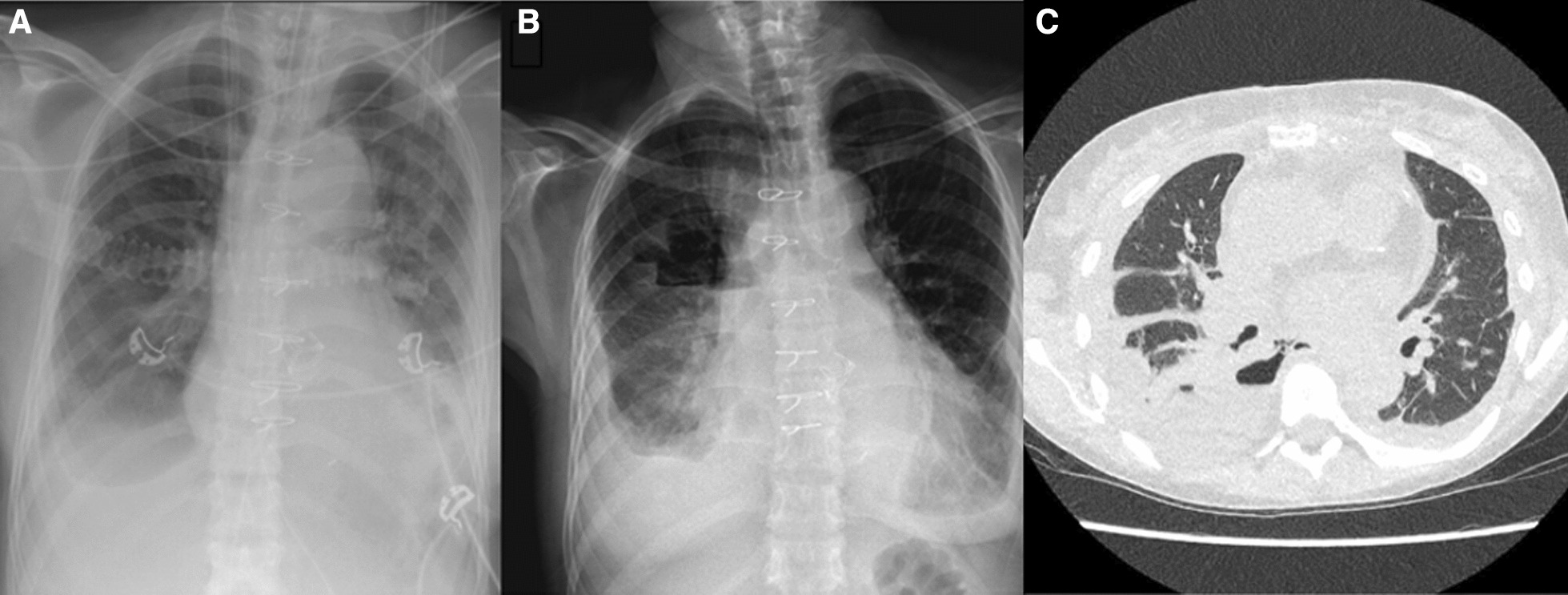
**A** postoperative chest X-ray on the ICU taken shortly after re-sternotomy because of the cardiac tamponade after AVR + CABG. **B** Regular control chest X-ray before discharge to referring hospital showing a new air-fluid level on the right side. **C** A coronal view of the CT-scan showing multiloculated air-containing pleural effusion strongly suspect of empyema

A 58-year-old woman was referred to our hospital because of symptomatic severe bicuspid aortic valve stenosis and a stenosis in the left anterior descending (LAD) coronary artery. She was electively admitted for surgical aortic valve replacement (SAVR) and coronary artery bypass grafting (CABG). Her medical history included hypertension, systemic lupus erythematosus, tertiary hyperparathyroidism, a Mallory Weiss lesion and a kidney transplant 15 years ago for which the patient had been using immunosuppressive medication (prednisone 5 mg per day and tacrolimus 3 mg per day) since. CABG (left internal mammary artery to LAD) and SAVR with a Carpentier-Edwards PERIMOUNT Magna Ease 21 mm biological prosthesis replacement was performed. Despite obvious risk factors for TEE, such as a previous Mallory Weiss lesion and chronic use of immunosuppressive medication, its use was deemed important for the intra-operative evaluation of cardiac and valvular function. During surgery, very fragile tissue was observed, conceivably due to many years of using immunosuppressive therapy. For example, the aortotomy required additional bovine pericardial patch repair during a second aortic cross-clamp time, due to tearing following initial routine closure by suture only. Direct postoperatively, a re-sternotomy for cardiac tamponade was performed during which TEE was again utilized. Both pleural cavities remained closed during the first and second procedure. Despite her frailty and a resternotomy, the patient was well enough to be transferred to the ward the following day. On the fourth day after surgery, the patient was recovering well without specific complaints. Routine postoperative investigations were performed as preparation for transfer to the referring hospital. However, chest radiography demonstrated an unexpected air-fluid configuration at the right side (Fig. 1A), which was new compared to the chest radiography 2 days earlier (Fig. 1B). Laboratory results showed c-reactive proteïn of 179 mg/L and leukocytes of 8.2 × 10^9^/L. Computed tomography (CT) demonstrated multiloculated air-containing pleural effusion, strongly suspect of empyema (Fig. 1C). Notably, the patient had no clinical symptoms of an empyema, normal dietary intake, with no fever and no respiratory complaints. CT-guided drainage of the fluid was performed for diagnostic purpose and showed red purulent fluid, with pH 5.4, amylase 49.470 U/L, lactate dehydrogenase 695 U/L and glucose 33.9 mmol/L. The pleural fluid was indicative for the presence of empyema and a decision was made for a surgical exploration of the right chest cavity.

**Fig. 2:**
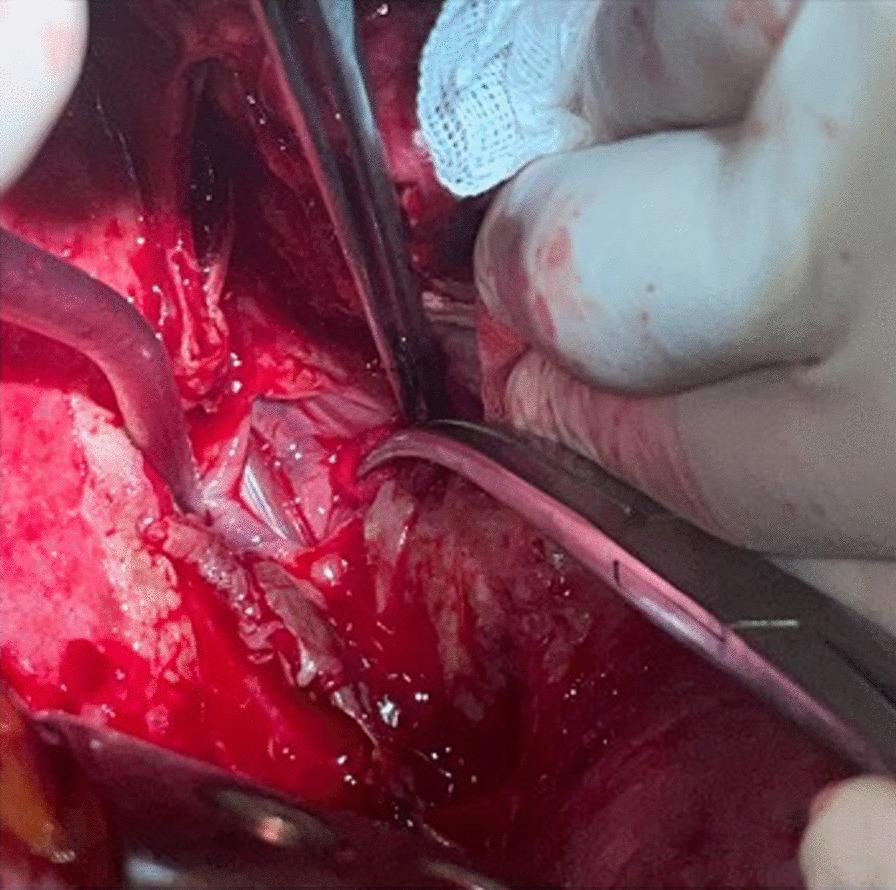
Intraoperative picture of the esophageal lesion also showing the gastric tube inside the esophagus

Video-assisted thoracic surgery of the right pleura was attempted, but was converted to a posterolateral thoracotomy due to dense adhesions. After extensive adhesiolysis, the right lung was mobilized to expose the mediastinum and perihilar region. Remarkably, pill medication was found in this region. Alongside these pills, a significant amount of debris was encountered, which we believe could possibly be remnants or altered forms of ingested food. Ultimately a tear of two by four centimeters in the mid-thoracic portion of the esophagus (Fig. 2). At this point, to facilitate the repair process by providing better orientation and identification of the esophagus, a nasogastric (NG) tube was inserted. The defect was primarily closed with a monocryl 4.0 suture in a two-layer fashion. Postoperatively, the NG tube was kept in place to provide gastric decompression and drainage, ensuring reduced strain on the repaired site.

Cultures of the empyema showed multiple microbes: Candida albicans, Citrobacter freundii, Streptococcus mitis, Streptococcus anginosus, Streptococcus epiderdimis, Prevotella species, Lactobacillus species, Eikenella corrodens and Neisseria subflava. The patient was treated with anidulafungin, meropenem and clindamycine. Postoperative course after the repair was uneventful. After a fasting period of 1 week, a barium swallow study was performed which showed no signs of a recurrent esophageal lesion. The patient was discharged 20 days after the surgical debridement of the empyema. At a follow-up appointment after 2 months, the patiënt was doing well and showed no signs of infection nor recurrent perforation.

## Discussion

This case describes the course of a patient after routine cardiacy surgery (SAVR + CABG) who developed an asymptomatic empyema due to an unnoticed esophageal injury caused by TEE. The incidence of esophageal perforation after TEE is reported between 0.03 and 0.09% [4]. In addition to gastrointestinal perforations, other complications can also occur as a result of TEE, such as dental injuries, lip compression, oropharyngeal injuries, dysphagia, odynophagia and gastrointestinal bleeding [1]. The overall complication rate due to TEE is considered low, with an incidence between 0.2 and 1.4% [1, 5]. However, patient-related risk factors have been identified predicting higher risk for TEE-related complications (Table 1), such as increasing age, low body mass index, previous cerebrovascular accident or transient ischemic attack, any previously reported abnormality of the esophagus, use of chronic immunosuppressive medication and previous GI bleeding [3]. Procedure-related risk factors have also been reported, including any cardiac procedure other than CABG, long cardiopulmonary bypass (CPB) time, return to operating room (OR) for any reason, higher activated clotting time during surgery, longer duration of intubation and longer TEE imaging time [3]. In the present case, it is likely that the risk was increased due to the chronic use of immunosuppresive medicine, previous Malory Weiss lesion and the need for resternotomy during which the TEE-probe was introduced a second time.

**Table 1:** Risk factors associated with TEE-related complications

Increased age [3, 5]
Low body mass index [5]
Previous CVA or TIA [3, 5]
Any previously reported abnormality of the esophagus [3]
Use of chronic immunosuppressive medication [3]
Previous GI bleeding [3]
Procedure other than CABG [5]
Longer CPB time [5]
Return to OR for any reason [5]
Higher ACT during surgery [3]
Duration of intubation [3]
Longer TEE imaging time [3]

In our opinion, in a patient with one or more risk factors for a TEE-related complication, the necessity of TEE should be critically reviewed and alternatives should be explored. The guidelines of the American Society of Anesthesiologist recommend that TEE may be used, if the benefit outweighs the potential risk, provided the appropiate precautions are applied. These precautions may include the following: consindering other imaging modalities (e.g., epicardial echocardiography), obtaining a gastroenterology consultation, using a smaller probe, limiting the examination, avoiding unnecessary probe manipulation and using the most experienced operator [6]. For the current case, in hindsight, TEE could have been omitted during the resternotomy. This particular case has created extra awareness in our team for a more restrictive use of TEE in patients with risk factors in procedures where TEE evaluation is not strictly necessary.

For an esophageal injury, different strategies can be considered. In this patient, the injury was not initially identified but was discovered during surgery to address the empyema. Given the circumstances, it was relatively straightforward to primarily close the defect with the thorax already open. However, if the esophageal injury is already known beforehand a multidisciplinary consultation and approach should be taken. Other treatment options such as stenting or secondary closure can be considered, depending on patient-specific factors.

Patients with an esophageal perforation and empyema usually report symptoms. Thoracic or abdominal pain is present in 70% of the patients, 44% of the patients have a fever and 26% have dyspnea. An empyema is estimated to occur in 8% of the patients with an esophageal perforation [7]. The patient described in the present case report did not have any symptoms at all, which is highly unlikely for an empyema due to an esophegael perforation. The only clue that led to the diagnosis was a routine chest X-ray showing a new air-fluid level which was very unusual since the pleural cavities had remained closed during both operations. There were no other signs for the perforation, particularly considering the fact that the chest X-ray taken 2 days earlier was unremarkable. A possible explanation is the chronic use of prednisone and tacrolimus that masked the symptoms of an empyema.

Brinster et al. showed that in 390 patients from 11 series, the overall reported mortality of esophageal perforation with treatment delayed by 24 h or more was 27% compared to 14% when treatment was initiated within 24 h [8]. The esophageal perforation of the patient described in this case report was discovered relatively late since there were no clear signs nor symptoms. Awareness and extra caution is needed in patients with risk factors, such as in our patient, to look for subtle signs of esophageal lesions. Early detection and prompt management of iatrogenic esophageal injuries are essential to prevent severe complications such as mediastinitis and sepsis, reducing the risk of long-term complications, and improving patient outcomes.

## Conclusion

TEE is a widely utilized and useful tool in cardiac surgery, the use of which rarely leads to complications. However, one should be aware that serious and life-threatening complications may occur, such as traumatic injury to the esophagus. The (previous) use of immunosuppressant medication is a risk factor for developing TEE-related complications and can additionally mask such serious complications. Therefore, the indication for TEE should always be critically appraised in patients using immunosuppressive medication, with additional focus during the postoperative course to detect possible masked complications timely.

## Data Availability

Data sharing is not applicable to this article as no datasets were generated or analyzed during the current study.

## Abbreviations

TEE: Transesophageal echocardiography
GI: Gastrointestinal
LAD: Left anterior descending
SAVR: Surgical aortic valve replacement
CABG: Coronary artery bypass grafting
CT: Computed tomography
NG: Nasogastric
